# Inulin as a Modulator of the Intestinal Barrier: Experimental Evidence, Mechanisms and Clinical Implications

**DOI:** 10.3390/biomedicines14040791

**Published:** 2026-03-31

**Authors:** Pablo Eliasib Martínez-Gopar, Fabiola Guzmán-Mejía, Marycarmen Godínez-Victoria, Jesús Flores-Valente, Daniel Efrain Molotla-Torres, Maria Elisa Drago-Serrano

**Affiliations:** 1 Departamento de Sistemas Biológicos, Universidad Autónoma Metropolitana Unidad Xochimilco, Calzada del Hueso No. 1100, Ciudad de México CP 04960, Mexico; pmartinezg@correo.xoc.uam.mx; 2 Sección de Estudios de Posgrado e Investigación, Escuela Superior de Medicina, Instituto Politécnico Nacional, Plan de San Luis y Díaz Mirón s/n, Ciudad de México CP 11340, Mexico; mgodinezv@ipn.mx (M.G.-V.); jesusfloresvalente@gmail.com (J.F.-V.); 3 Doctorado en Ciencias Biológicas y de la Salud, Universidad Autónoma Metropolitana Unidad Xochimilco, Calzada del Hueso No. 1100, Ciudad de México CP 04960, Mexico; efrainmolotla@gmail.com

**Keywords:** inulin, dietetic fiber, intestinal permeability, gut barrier, tight junction proteins, fructooligosaccharides

## Abstract

The intestinal epithelial barrier regulates paracellular transport, and its dysfunction is associated with inflammatory and metabolic diseases. Among dietary fibers, inulin has attracted considerable attention due to its beneficial effects on intestinal health. Inulin’s actions have been attributed to protecting the structure and function of gut barrier components against inflammatory-associated damage. This review integrates preclinical and clinical studies evaluating the impact of inulin on intestinal permeability. Evidence from in vitro and in vivo models shows that inulin regulates the expression of tight junction proteins (TJPs), Paneth cell proliferation, and antimicrobial peptides, and modulates inflammatory signaling pathways. In addition, inulin prebiotic activity, via microbiota, stimulates the production of short-chain fatty acids (SCFAs) as butyrate that reinforces the barrier function. Understanding these pathways highlights the therapeutic potential of inulin as a nutritional strategy for treating barrier dysfunction. Clinical studies in obesity, metabolic disorders and inflammatory intestinal disease have associated inulin supplementation with improvements in biomarkers of intestinal permeability. Future studies are needed to test inulin’s safety in order to prevent potential risks and hazards.

## 1. Introduction

Gut barrier components including microbiota, mucus, epithelium and the subepithelial mediators of intestinal immunity are responsible for intestinal homeostasis, referring to a balanced condition, resulting from the capacity of the intestinal barrier to preserve its functional and structural integrity against adverse conditions. The prime function of the intestinal barrier is selective transport that consists in allowing the entry of innocuous molecules like nutrients, water, and vitamins and, at the same time, blocking the transport of potentially harmful agents like toxins to the inner milieu [1]. This dichotomous function is orchestrated through the complex interplay of a wide array of signal transduction pathways of the gut epithelial monolayer with microbiota, the mucus layer and the subepithelial components of the intestinal immunity [2].

Intestinal epithelium consists of a monolayer of cells connected collaterally to each other via the TJPs that determinate epithelial polarization, which entails an apical (luminal) membrane in contact with the intestinal lumen and paracellular (basolateral) membrane orientated to the lamina propria. Tight junction protein complexes have a critical role in regulation of paracellular transport, which refers to the passive diffusion of solutes through the space between the paracellular membranes of the adjacent cells [3].

Gut barrier paracellular permeability can be disturbed under inflammatory conditions; therefore, plant-derived polysaccharides such as inulin have long been a focus of research due their regulatory impact on intestinal permeability [4].

Inulin is a plant-derived non-digestible fiber that acts as a prebiotic because it is not degraded by host intestinal enzymes, but rather by enzymes derived from colonic microbiota [5]. Inulin displays beneficial outcomes on gut barrier function after being fermented by gut microbiota to render SCFAs [4,6]; furthermore, inulin shows microbiota-independent effects by attenuating the impact of lipopolysaccharide (LPS)-induced inflammation on tight junction protein disassembly and on increased intestinal permeability [7].

This review integrates current experimental and clinical evidence describing how inulin modulates intestinal epithelial barrier function. We discuss mechanisms of inulin’s regulation of gut barrier components and inflammatory signaling pathways. Finally, the clinical relevance of these mechanisms is examined in the context of metabolic and inflammatory disease associated with intestinal barrier dysfunction.

## 2. Gut Permeability Overview

### 2.1. Components of Intestinal Epithelial Monolayer

Intestinal epithelium consists of a single layer of cells raised from LGR5+ multipotential stem cells located at Lieberkühn’s crypts that, whilst climbing until reaching the villus tip, undergo full differentiation, rendering mature cells including enterocytes, Paneth cells, goblet cells, enteroendocrine cells, tuft cells and M cells [8].

The epithelial monolayer accomplishes the transport of solutes via transcellular and paracellular mechanisms [9]. Paracellular transport refers to the passive diffusion of solutes across the space between the paracellular membranes of the adjacent cells. The epithelial monolayer can be categorized as “leaky” epithelia when the ion conductivity is higher in the paracellular pathway than the transcellular pathway, and this pattern predominates in the small intestine; conversely, in “tight” epithelia, the ion conductivity is higher in the transcellular pathway than the paracellular pathway that predominates in the colon [10]. Paracellular transport entails (i) a pore-forming pathway for the selective permeation of 5–10 Å solutes, mainly ions and small uncharged solutes; (ii) a leaky pathway for non-selective transport of <100 Å large-size solutes like large ions and molecules irrespective of their charge; and (iii) an unrestricted pathway that allows the permeation of solutes regardless of their size and/or charge [3].

Epithelial monolayer cells are connected adjacently to each other through junctional protein complexes, namely adherence junctions, desmosomes and TJPs [11]. Adherence junctions are expressed at the middle of the basolateral membrane, and they stabilize the cell–cell interaction contacts through the transmembrane proteins E-cadherin and nectin, connected to the actin cytoskeleton via the cytoplasmic protein catenin or afadin, respectively [12]. Desmosomes are expressed at the extreme of the basolateral membrane, and they provide mechanical strength to the intercellular cell–cell contact during peristalsis and include the transmembrane proteins desmoglein and desmocollin, linked to intermediate filaments through the cytoplasmic proteins plakophilin, desmoplakin and plakoglobin [13].

The TJP complex determinates the polarization of the epithelial membrane in the apical membrane, facing the lumen, and the basolateral membrane, orientated to both the lamina propria and the intercellular membrane spaces between adjacent cells; in fact, the TJP complex is expressed at the most apical extreme of the basolateral membrane [10]. Tight junction proteins like claudins regulate paracellular transport via the pore-forming pathway for the selective permeation of 5–10 Å solutes, mainly cations, while occludin and zonula occludens (ZO-1) control the leaky pathway for non-selective transport of <100 Å large-size ions and solutes regardless of their charge [3]. Paracellular transport can be expressed in quantitative terms as “intestinal permeability” that measures the rate of transport across the epithelial cell monolayer by several assays [14]. The TJP complex includes claudins, occludin, tricellulin and junctional adhesion molecule A (JAMA). These proteins are anchored to the peri-junctional actomyosin ring via scaffolding intracellular proteins such as ZO-1 protein, afadin and cingulin [15,16]. The peri-junctional actomyosin ring is a ring of myosin and actin just below the TJP complex that has a determining role in the structural organization and stabilization of the TJP complex [17].

Claudins encompass a family of tetraspan transmembrane proteins that exhibit two extracellular loops and a short N-terminal and long C-terminal intracellular domains. Claudin extracellular loops form selective channels that stablish either homophilic interactions among the identical claudins or heterophilic ones among different claudins on adjacent cells [18]; otherwise, claudin subunits form cis-interactions (intracellular) within the same cell membrane and trans-interactions (intercellular) in claudins expressed on adjacent cells [18]. The intracellular C-terminal domain determinates claudins’ link with the PDZ-binding domain of the scaffolding ZO proteins. Claudins have a critical role in the regulation of paracellular permeability and functionally, and they can act as pore-sealing agents, which enforce paracellular sealing, and pore-forming claudins, which weaken the paracellular interaction; some claudins have undefined roles [10,19].

Occludin belongs to the tight-junction-associated Marvel domain-containing proteins (TAMP) that contain four transmembrane domains with a short cytoplasmic N-terminal domain and a long cytoplasmic C-terminal domain. The intracellular C-terminus moiety interacts with the PDZ domain of ZO-scaffolding proteins. The homophilic interaction between the extracellular occludin domains of adjacent cells allows the permeation of cations but hampers the permeation of macromolecules. Occludin in phosphorylated form contributed to TJ assembly and paracellular permeability regulation [20]. Tricellulin, also known as MarvelD2, belongs to the TAMP family and it is structurally related to occludin that exhibits two extracellular loops, four transmembrane domains, a cytoplasmic C-terminal domain and a cytoplasmic N-terminal domain [17]. Homophylic interactions of the extracellular domains of tricellulin in adjacent cells act as barriers for macromolecules. Both cytoplasmic N and C-terminal loops of tricellulin seem to play a relevant role in tricellulin localization at the tight junctional hub. Unlike occludin that can be found in both bicellular and tricellular tight junctions, tricellulin is localized primarily in tricellular junctions where it acts as a regulator of the leaky pathway. Tricellulin is localized prominently in the small intestine and has a role in tight junction reassembly and barrier development [15,17].

Junctional adhesion molecule A (JAMA) is located at epithelial and endothelial tight junctions. JAMA is a transmembrane member of the immunoglobulin superfamily, containing two extracellular immunoglobulin-like N-terminal domains, a single transmembrane and a cytoplasmic C-terminal loop containing a PDZ domain [21]. Extracellular domains are involved in cell-to-cell contact, cell migration and transcytosis. JAMA forms either cis-homophylic dimers between adjacent cells or trans-homophylic dimers in opposite cells. The cytoplasmic loop contains potential sites of phosphorylation and a C-terminal PDZ-domain motif that enables the interaction with scaffolding intracellular proteins such as ZO proteins, afadin and cingulin, among others [21]. Phosphorylation of JAMA at the cytoplasmic C-terminal loop is required for the structural stabilization, maturation and functionality of tight junctions [21].

Zonula occludens (ZO) proteins are scaffolding peripheral proteins located immediately below the tight junction membrane contact points, and ZO-1 represents the member most known. ZO proteins contain PDZ domains involved in the interaction with the C-terminal cytoplasmic domain of transmembrane proteins claudins, occludin, JAM-A and tricellulin. The C-terminal domain of ZO proteins also interacts with adaptor and scaffolding proteins that lack PDZ domains like cingulin and paracingulin and actin [10,16,22]. Zonula occludens proteins have an essential role in the regulation of claudin polymerization in epithelial cells, as well as in tight junction stabilization and function. Furthermore, ZO proteins are involved in signal transduction and in the regulation of transcription [22].

Afadin, also termed AF-6, is a scaffolding protein containing several protein-binding domains, including a PDZ domain that enables its ligation to nectin, E-cadherin, JAMA, ZO proteins and claudins; afadin presents one canonical isoform characterized by three prolin-rich domains at the C-extreme where the site of F-actin binding lies; the non-canonical s-afadin isoform lacks an F-actin binding site [15]. Afadin is expressed at both tight junction and adherence junction protein complexes and acts as a link between apical junctional complexes and the intracellular components of the cytoskeleton and contributes to preserving the integrity of intestinal barrier function [15,21].

Cingulin is a non-PDZ cytoplasmic scaffolding protein associated with tight junction and adherence junction protein complexes. Cingulin is a homodimer with an N-terminal globular head region and a long α-helical coiled rod and small globular tail. The N-terminal head region interacts with ZO-1, JAMA and actin, while the long α-helical coiled region enables the cingulin–cingulin interaction [15]. The role of cingulin in intestinal permeability is uncertain, but its downmodulation is related to impaired barrier formation; moreover, cingulin is involved in signaling pathways that regulate the expression of claudin-2, a pore-forming claudin [15].

Accordingly, the TJP complex—with a critical role in intestinal permeability regulation—is a target for a wide array of molecules, such as inulin, as described in the following sections. An overview of signal pathways that regulate the expression and localization of TJPs is described next.

### 2.2. Inflammatory Pathways Modulating Intestinal Permeability

Several pathological and physiological conditions, including obesity, intestinal inflammation, and psychological stress, are recognized as potent inflammatory stimuli that compromise intestinal barrier integrity. These conditions promote the release of pro-inflammatory cytokines and activation of signaling pathways that disrupt the structural organization and function of tight junction complexes, leading to altered expression and redistribution. As a result, epithelial permeability increases, facilitating the translocation of luminal antigens and microbial products that further exacerbate mucosal inflammation and barrier dysfunction [23,24,25,26].

**Figure 1:**
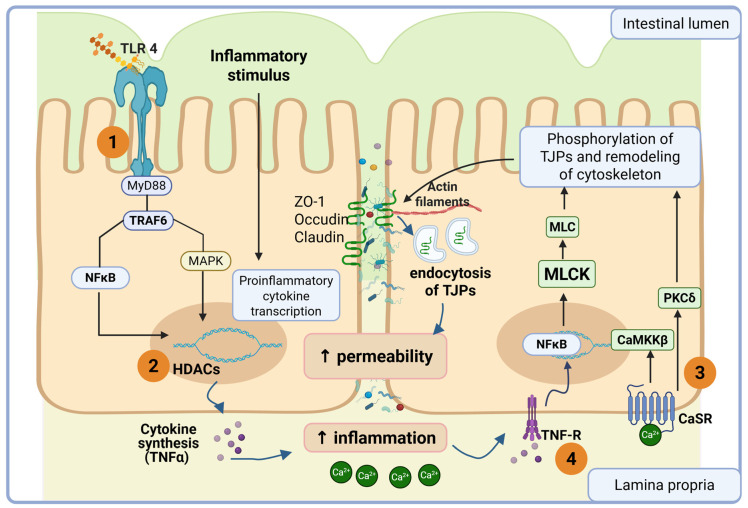
Overview of inflammatory signal pathways. (1) LPS-TLR 4 interaction drives transcriptional factor activation, such as nuclear factor κB (NFκB) and mitogen-activated myosin kinase (MAPK); both enable the expression of pro-inflammatory cytokines including tumor necrosis factor α (TNF-α). (2) Histone deacetylases (HDACs) remove acetyl groups from histones, promote chromatin condensation, and repress the expression of genes involved in tight junction maintenance. (3) Inflammation-induced increase in intracellular calcium activates Calcium/Calmodulin-dependent Protein Kinase Kinase-β (CaMKKβ) and Protein Kinase Cδ (PKC-δ) that lead to contraction of the peri-junctional actomyosin ring and endocytosis of tight junction complexes; this remodeling results in opening of spaces between the adjacent membranes of the epithelial monolayer, causing a paracellular permeability increase. (4) Through its ligation with TNF Receptor (TNFR), TNF-α induces Myosin Light Chain Kinase (MLCK) transcription via NFκB activation. MLCK phosphorylates Myosin Light Chain (MLC), leading to contraction of the peri-junctional actomyosin ring and endocytosis of tight junction complexes, causing a paracellular permeability increase. Created in BioRender. Guzman, F. (2026) https://BioRender.com/sv4w8vb (accessed on 23 March 2025).

The following inflammatory signal pathways involved in the intestinal permeability are described next and depicted in the Figure 1.

Microbial products such as lipopolysaccharide (LPS) activate pattern-recognition receptors including Toll-like receptor 4 (TLR 4), initiating intracellular signaling through Myeloid differentiation primary response 88 (MyD88) and tumor necrosis factor receptor-associated factor 6 (TRAF6). This cascade activates nuclear factor κB (NFκB) and mitogen-activated myosin kinase (MAPK) that, in turn, enhance the transcription of pro-inflammatory cytokines such as tumor necrosis factor α (TNF-α) that contribute to epithelial barrier dysfunction [27]. In addition to transcriptional regulation, inflammatory signaling can influence epigenetic control of barrier-associated genes through the activity of histone deacetylases (HDACs), which remove acetyl groups from histones, promote chromatin condensation, and repress the expression of genes involved in tight junction maintenance. These inflammatory mediators further destabilize epithelial junctions by promoting cytoskeletal remodeling and the endocytosis or redistribution of junctional proteins associated with the actin cytoskeleton [28,29]. Concurrently, inflammation favors calcium intracellular transport via calcium sensor receptor (CaSR); intracellular Ca^2+^ level increase in epithelial cells activates calcium/calmodulin-dependent protein kinase kinase β (CaMKK-β) and protein kinase Cδ (PKC-δ); both kinases cause TJP phosphorylation, leading to concomitant cytoskeleton remodeling, which results in opening of spaces between the adjacent membranes of the epithelial monolayer, causing a paracellular permeability increase. MLCK phosphorylates the myosin regulatory light chain, leading to contraction of the peri-junctional actomyosin ring and structural remodeling of tight junction complexes. Through its ligation with TNF receptor (TNFR), TNF-α induces Myosin light Chain Kinase (MLCK) transcription via NFκB activation. MLCK phosphorylates myosin light chain (MLC), leading to contraction of the peri-junctional actomyosin ring and endocytosis of tight junction complexes, resulting in an increase in intestinal permeability [28,29].

Collectively, these inflammation-driven transcriptional, epigenetic, and cytoskeletal mechanisms increase paracellular permeability and contribute to the amplification of the intestinal inflammatory response.

## 3. Inulin Overview

Dietary fiber derived from plants is a type of carbohydrate that cannot be digested by the body, which mainly includes cellulose, pectin, gums, resistant starch, lignin and fructans [30]. Fructans are a large group of carbohydrates composed primarily of repeating fructose units linked by linear bonds (β-2,1), attached to a terminal glucose molecule [31]. Among fructans there are different types according to their degree of polymerization (DP), so they can be classified into short-chain fructans (2–5), known as short-chain fructoligosaccharides (sc-FOS), or long-chain fructans (>60) [31].

Inulin is a type of fructan, a naturally occurring polysaccharide produced by a variety of plants including the cotyledonous family, such as *Amaryllidaceae*, *Compositae*, *Gramineae* and *Liliaceae* [31]. Some sources of inulin are Jerusalem artichoke, chicory, garlic, raw asparagus, salisfy, yacon, and onion, among some others [31].

Structurally, inulin molecules are linear polysaccharides composed of fructose units, generally 2–60, linked by β-2,1 D-fructofuranose bonds, with a glucose residue at the end [30]. Depending on the degree of polymerization, inulin can be divided into short-chain inulin (DP 2–10), known as fructooligosaccharides (FOS), and long-chain inulin (DP 10–60) [30,31]. Oligofructose is extracted from plants and can also be produced by partial enzymatic hydrolysis of inulin. Variations in DP of inulin depend on various factors, such as the plant sources, the maturity of the plant, the method of extraction, and storage, among others. Therefore, the DP determines different physicochemical properties and will influence inulin’s functionality [30,32]. Thus, the sweetness of FOS is mild, due to its higher monosaccharide and disaccharide content, while long-chain inulin is only slightly sweet. Thus, inulin is used as a substitute for sucrose in various food products.

The solubility of long-chain inulin in water is low (about 10% at 25 °C) but increases with temperature, reaching up to 33% at 90 °C. Furthermore, FOS is more soluble in water than long-chain inulin, which is attributed to its lower degree of polymerization, higher proportion of small molecules (mono- and disaccharides), and more intense interaction with water via hydrogen bonds [30,32].

Inulin has high gelling properties and forms a viscous structure when completely dissolved in water or any other aqueous medium, forming a white, creamy structure that can be easily added to foods as a fat substitute, mainly in dairy products [32].

These physicochemical properties have allowed inulin to be used as an additive in many food products, to improve flavor and texture and reduce the amount of sucrose or fat used in their production. Moreover, inulin stands out for its prebiotic properties for *LactobacilIus* and *Bifidobacteria*, positioning it as a naturally sourced product that can help improve intestinal health by decreasing the colonization of harmful microflora.

Prebiotics are defined by the International Scientific Association for Probiotics and Prebiotics (ISAPP) as “a substrate that is selectively utilized by host microorganisms conferring a health benefit” [33]. These are the fermentable ingredients capable of producing definite changes in activity as well as compositions of gastrointestinal microflora including *Lactobacillus* and *Bifidobacteria*.

Inulin is a dietary fiber with a potential prebiotic effect. Inulin fermentation occurs entirely in the colon through an anaerobic process mediated by microbiota that produces gases such as carbon dioxide, hydrogen, methane, and organic acids such as lactic acid and SCFA [34]. The SCFAs produced by colonic fermentation of inulin are mainly acetic acid, propionic acid and butyric acid. Some human studies have shown that fermentation of inulin, administered as dietary fiber, induces an increase in butyrate concentration that is related to increased growth of *Lactobacillus* or *Bifidobacteria* strains [35,36,37]. Furthermore, the SCFAs generated by inulin fermentation also act as signaling molecules that bind to receptors on enteroendocrine cells, increasing the production of postprandial intestinal hormones, which are involved in regulating metabolism, glucose and body mass [38]. Thus, inulin, as a dietary fiber, can improve the metabolic profile in diseases such as obesity and diabetes [35,36,37]. Also, the increased production of SCFAs lowers colon pH, which prevents the growth of pathogenic bacteria.

Moreover, SCFAs not only act as an energy source for colonocytes, also play an immunomodulatory role through receptors such as G protein receptors (GPR) GPR41, GPR43, and GPR109A. The signaling pathways powered by interaction of SCFAs with their receptors promote, in the intestinal epithelium, favorable morphological changes, for example, increasing villi height, deepening crypts, and elevating the number of goblet cells. It also thickens the mucus layer in the colon, which strengthens the barrier against harmful events that can damage it [39]. This epithelial remodeling is partially mediated by signals from the immune system, which promote the proliferation of LGR5+ intestinal stem cells and the expression of genes associated with mucus production. Furthermore, the presence of functional microbiota is essential for these structural effects: in bacteria-free or antibiotic-treated models, the changes induced by inulin are attenuated or disappear [40]. In vitro studies using Caco-2 cells have reported that LPS treatment increased the paracellular permeability of the cell monolayer, demonstrating a deterioration of the intestinal barrier, which inulin attenuates [7]. These findings suggest that inulin may regulate the expression of TJPs and preserve the integrity of the intestinal barrier under pro-inflammatory stimuli. These results, raised in cell cultures, indicate actions of inulin independent of the intestinal microbiota.

However, the effect of inulin on the intestinal barrier is not always beneficial. Under conditions of induced dysbiosis, such as after antibiotic treatment, inulin supplementation can delay mucosal immune recovery, elevate serum LPS levels, and increase diamine oxidase (DAO) activity, indicative of impaired intestinal permeability [41]. Furthermore, in rodent models of Dextran Sodium Sulfate (DSS)-induced colitis, inulin administration exacerbates the severity of inflammatory damage [42]. Data suggested that the preexisting immunological and inflammatory environment influences inulin’s outcome to be either beneficial or harmful.

Thus, the findings described above indicate that inulin can strengthen the intestinal barrier through mechanisms involving microbiota modulation, stimulation of epithelial reparative capacity, and regulation of inflammation via the immune system. However, its effect depends on the state of the intestinal microbiota and the host’s immune profile, as it could have adverse effects under conditions of dysbiosis or active inflammation.

Mechanisms and methods to assess intestinal permeability are applied for basic studies in animal experimentation and clinical trials in humans, as mentioned in the following.

## 4. Effects of Inulin on Intestinal Permeability: In Vitro and In Vivo Assays

Inulin and its derivatives have emerged as effective dietary agents for mitigating intestinal barrier permeability dysfunction in several models of local or systemic inflammation. Both in vivo and in vitro models have demonstrated the protective effect of inulin and its derivatives on microbiota-dependent and -independent mechanisms.

### 4.1. In Vitro Studies

In vitro studies, using monolayers of human intestinal epithelial cells (such as Caco-2 or T84), provide relevant information on the direct action (microbiota-independent) of inulin. For this purpose, techniques such as Trans-Epithelial Electrical Resistance (TEER), Lucifer Yellow (LY) paracellular flow and Fluorescein Isothiocyanate-labeled Dextran assay (FITC-Dextran) are often used. TEER is a technique that measures the electrical resistance of a cultured cell monolayer, being a direct indicator of the integrity of the tight junctions. TEER is performed by applying an electric current through the monolayer. A higher TEER correlates with lower permeability since resistance reflects cell junction strength. LY measures the movement of the fluorescent dye through the space between cells (paracellular pathway). LY is a small molecule that can only pass when tight junctions are open or compromised. A lower flow of LY across the monolayer indicates an intact and less permeable barrier [43]. FITC-Dextran is also used to measure paracellular permeability. The 4 kDa dextran is the most used; the small size allows it to cross altered cell barriers but not intact ones, and the fluorescent marks allow visualization of this passage through barriers [7].

#### 4.1.1. Basal Conditions

There is a disagreement on the effect of inulin on permeability observed in unprimed Caco-2 cell cultures; some studies have not found direct effects of inulin via TEER [43,44]. However, high concentrations of FOS significantly decreased TEER in a dose-dependent and reversible manner [45]. FOS-induced TEER decrease reflects the increase in paracellular permeability, which might be a physiological mechanism for improving calcium ion (Ca^2+)^ absorption.

#### 4.1.2. Chemical Insults

It has been observed that the chain length of fructans is a determining factor in protection against barrier-disrupting agents. In T84 cells, inulin was more effective in maintaining TEER against damage induced by phorbol 12-myristate 13-acetate (PMA) than FOS. PMA is an ester that causes alterations in permeability through the activation of Protein Kinase C (PKC) [46]. In T84 cells, chicory inulin counteracted the TEER-induced decrease caused by the calcium ionophore A23187 [47]. This suggests that inulin may have a direct stabilizing effect on cell membranes, independent of microbial fermentation and depending on the chain length.

#### 4.1.3. Microbial Challenges

The protective effect of inulin on permeability has also been observed against biological stimuli. In T84 cells, chicory inulin prevents the TEER-induced decrease caused by the food-contaminant mycotoxin Deoxynivalenol (DON) [47]. In Caco-2 cells, inulin pretreatment mitigated the LPS-induced increase in paracellular permeability, assessed by FITC-Dextran diffusion across the cell monolayer [7]. In a Caco-2 Bbe1 cell culture, inulin and FOS attenuated the decline in TEER and the increase in FITC-Dextran in cells infected with enterohemorrhagic *Escherichia* (*E.*) *coli* O157:H7 (EHEC) [48].

All experimental evidence indicates that the microbiota-independent effect of inulin on intestinal permeability is determined by the inulin concentration, the inulin chain length, and the applied challenge. The key findings from in vitro experiments regarding the impact of inulin and its derivatives on intestinal barrier function are summarized in Table 1, detailing the specific models, stimuli, and dosages employed.

Evidence from in vitro models indicates a non-dependent effect of inulin on intestinal permeability, where basal integrity remains unaffected by low doses but improves significantly at concentrations between 10% and 15% *w*/*v*. However, FOS supplementation (100 mmol/L) may paradoxically compromise membrane stability, highlighting a critical therapeutic window. In the presence of chemical or biological insults, the protective range expands considerably, demonstrating efficacy across a broader spectrum of concentrations. Furthermore, while FOSs show specific localized protection against certain chemical stressors, inulin appears more robust against a wider variety of biological challenges, though the current scarcity of comparative studies precludes a definitive consensus on chain-length superiority. Temporal dynamics are equally essential, with established protective effects requiring incubation periods of 16 to 24 h, whereas shorter exposures (under 6 h) fail to provide benefits and may even exacerbate permeability. Thus, maximizing the in vitro efficacy of fructans necessitates precise calibration of concentration, molecular structure, and exposure duration to avoid adverse outcomes and ensure optimal barrier reinforcement.

### 4.2. In Vivo Studies

Multiple animal model studies have used FITC-Dextran to assess intestinal paracellular permeability. In this in vivo method, FITC-Dextran is administered orally. When the intestinal barrier is compromised, FITC-Dextran is translocated into the bloodstream, and its concentration is measured in plasma or serum to quantify permeability [50]. The measurement in urine of non-digestible sugars such as sucrose, lactulose and mannitol is also used to assess intestinal permeability. A sucrose test is used to measure permeability in the stomach while, a lactulose: mannitol test can be used to measure small intestine permeability [51]. Blood levels of endogenous molecules, such as LPS, LPS-binding protein (LBP), D-lactate (D-lac), and p-Cresyl (PCS), can be used to determine intestinal barrier permeability. LBP, a marker of bacterial translocation, indicates that LPS has crossed the intestinal barrier [52] and, eventually, LPS reaches the blood stream, causing endotoxemia. D-lac is a bacterial metabolite found in the intestine [53], and PCS is a gut-derived uremic toxin [54], whose detection in systemic circulation indicates intestinal barrier impairment.

#### 4.2.1. Basal Conditions

Experimental settings have addressed impact of inulin on intestinal permeability under basal conditions. In healthy Sprague-Dawley rats, inulin did not alter either sucrose: mannitol or lactulose: mannitol tests [51]. However, in healthy BALB/c mice, inulin decreased the plasma FITC-Dextran levels [50]; in addition, in healthy Kunming mice, inulin increased TJP levels [55]. The variability observed in the effects of inulin on intestinal permeability can be attributed to the sensitivity of the methods used. Sucrose and lactulose have a low molecular weight and are used for the detection of subtle alterations in permeability; on the other hand, FITC-Dextran is a larger macromolecule used for identifying serious alterations in the integrity of the epithelial barrier.

#### 4.2.2. High-Fat Diets

Inulin has shown beneficial results in modulating intestinal permeability in high-fat consumption models. In mice subjected to a high-fat diet (HFD), inulin prevented the increase in plasma levels of LBP [52] and favored the increase in transmural resistance ex vivo assessed by Ussing chambers [56]. The Ussing chamber is used to measure the transport of ions, nutrients, and drugs across various epithelial tissues. Additionally, in germ-free mice with severe acute pancreatitis induced by HFD, inulin also reduces the levels of LPS and D-lac in plasma [53]. The decrease in D-lac after inulin supplementation confirms the restoration of intestinal permeability. These results show that inulin improves intestinal permeability in HFD models in a microbiota-dependent and -independent way.

#### 4.2.3. Acute Inflammation

In experimental models of acute inflammation, including bacterial infection and food allergy, inulin shows different effects on permeability. In a *Citrobacter rodentium* infection model, inulin and FOS failed to reduce the infection-induced increased intestinal permeability as measured by FITC-Dextran [49]. In a food allergy model, a medium dose of inulin was effective in counteracting the decrease in TJPs and the increase in LPS in serum, whereas low and high doses of inulin aggravated the histopathological damage in the intestine and increased the serum LPS concentration [57]. These effects of inulin appear to depend on the stimulus that causes intestinal permeability dysfunction and the doses applied.

#### 4.2.4. Chemical Compounds

Inulin has been shown to be a mitigator of hyperpermeability induced by various chemical compounds. In mice treated with ketamine, inulin decreased the serum and colonic LPS levels [58]. In this context, in the offspring of mice perinatally exposed to environmental pollutants such as Perfluorooctanoic Acid (PFOA) or Ammonium perfluoro (2-methyl-3-oxahexanoate) (GenX), inulin significantly decreased serum FITC-Dextran concentrations, indicating a reduction in intestinal permeability [59]. Modulation of the maternal gut microbiota has been proposed as the mechanism behind the protective effect. This maternal microbial modulation allows offspring to have a healthier intestinal microbiota, thus resulting in strong epithelial development to face environmental pollutants [59].

#### 4.2.5. Neurobehavioral Disorders

The TLR 4 receptor appears to be a key point in the effects of inulin on intestinal permeability, not only after exposure to chemicals but also in stress models. Inulin inhibited stress-induced alterations in intestinal barrier integrity in mice, reducing serum LPS levels [60]. Although inulin shows beneficial effects on permeability in the unpredictable chronic stress model, inulin did not change FITC-Dextran levels in BTBR mice, which are used as a model of autism spectrum disorder. This difference in the effects could be due to the treatment duration; in the unpredictable chronic stress model, nine weeks of supplementation showed positive effects, while in the autism spectrum disorder model, three weeks was insufficient to show any beneficial effect.

#### 4.2.6. Systemic Pathologies

Finally, the effect of inulin on intestinal permeability has been studied in other systemic pathologies such as abdominal aortic aneurysm and chronic kidney disease. In mice with abdominal aortic aneurysm (AAA), a condition associated with chronic inflammation, an inulin diet effectively reduced plasma FITC-Dextran fluorescence intensity, suggesting decreased permeability [61]. In adenine-induced chronic kidney disease in Sprague-Dawley rats, inulin reduced the levels of PCS in serum, suggesting a beneficial effect on intestinal permeability [54]. The protection of the intestinal barrier in these models highlights the therapeutic potential of inulin in systemic diseases that affect the intestinal barrier.

According to the above, experimental evidence indicates that the protective effect of inulin is strongly dependent on the administered doses, the treatment time, the animal model, and the evaluation method; therefore, the evaluation of inulin’s impact on intestinal permeability must consider these technical aspects. Table 2 summarizes the results of inulin supplementation in animal models, including treatment protocols and their subsequent impact on intestinal barrier markers.

In summary, in vivo studies provide evidence about the microbiota-dependent impacts of inulin on intestinal permeability; on the other hand, in vitro studies have been critical to study the microbiota-independent mechanisms induced by inulin in depth. All these mechanisms are explored in the next section.

The biological efficacy of inulin on intestinal barrier integrity is fundamentally governed by dosage, molecular chain length, and intervention duration. While basal conditions require higher concentrations—typically exceeding 10% *w*/*w* or 2.5 g/kg—to enhance TJP expression, lower doses demonstrate significant protective capacity primarily under pathological challenges such as high-fat diets or systemic inflammation. Furthermore, structural characteristics play a decisive role; inulin consistently outperforms FOS in mitigating permeability alterations across diverse disease models. Finally, the evidence underscores a temporal requirement for success, suggesting that sustained supplementation for at least four to nine weeks is necessary to consolidate epithelial defenses, whereas short-term protocols remain largely ineffective except in specific hypersensitivity contexts. Consequently, optimizing the therapeutic application of inulin requires a strategic alignment of these three variables to ensure robust physiological protection.

### 4.3. Mechanisms of Inulin’s Action on Permeability

The effects of inulin on intestinal permeability have been focused on two presumable mechanisms that are interconnected and act synergistically in restoring the barrier function that are described below.

#### 4.3.1. Mechanisms Related to Gut Barrier Components

The beneficial effects of inulin on intestinal homeostasis are largely mediated by its interaction with the gut microbiota and the metabolites generated during its fermentation. In several murine models, inulin supplementation has been shown to enrich beneficial bacterial populations, including *Akkermansia*, *Bifidobacterium*, *Ileibacterium*, and *Muribaculum*. In AAA mice and hepatic steatosis rat models, both inulin and FOS increased the abundance of *Akkermansia muciniphila*, which was associated with improvements in the integrity and thickness of the intestinal mucus layer [54,56]. The mucus layer represents the first line of physical and chemical defense, trapping microorganisms and toxins and preventing their direct contact with the epithelial surface [2].

Consistent with these observations, inulin supplementation has also been shown to reverse intestinal dysbiosis induced by chronic administration of ketamine or a high-fat diet (HFD), restoring microbial balance and increasing the abundance of butyrate-producing bacteria [45,51]. The fermentation of inulin by the gut microbiota results in the production of short-chain fatty acids (SCFAs), including acetate, propionate, and particularly butyrate, which contribute to the maintenance of intestinal barrier integrity [46,54,56].

SCFAs represent the main source of energy for colonocytes, covering up to 70% of their energy requirements. This metabolic contribution supports epithelial renewal, proliferation, and functional recovery of the intestinal barrier, as documented in rodent models of hepatic steatosis in Sprague–Dawley rats and healthy Kunming mice [48,56]. However, the effects of inulin on SCFA production may vary depending on host physiology and microbiota composition. For example, in C57BL/6 mice infected with EHEC, inulin supplementation increased acetate levels without significantly altering other SCFAs [42], whereas in healthy wild-type C57BL/6 and germ-free mice no significant changes in SCFA production were observed [57].

After microbial fermentation, butyrate can enter colonocytes by passive diffusion or through transporters such as the H^+^-coupled monocarboxylate cotransporter (MCT1), where it is oxidized in the mitochondria to generate ATP via the respiratory chain [58]. Beyond its metabolic role, butyrate also functions as a key signaling molecule that regulates several components of the intestinal barrier. For instance, butyrate promotes goblet cell proliferation and increases the expression of MUC2, the principal mucin of the colonic mucus layer [63], thereby enhancing mucus layer integrity. In epithelial cells, butyrate strengthens barrier function through multiple complementary mechanisms, including inhibition of histone deacetylases (HDAC) [64], activation of short-chain fatty acid-sensing G protein-coupled receptors, and stimulation of AMPK signaling [65]. These pathways collectively promote TJP expression, relocalization, and assembly, leading to improved epithelial cohesion and barrier stability. In addition, butyrate may stimulate Paneth cells to increase the production of antimicrobial peptides, further contributing to mucosal defense [66].

**Figure 2:**
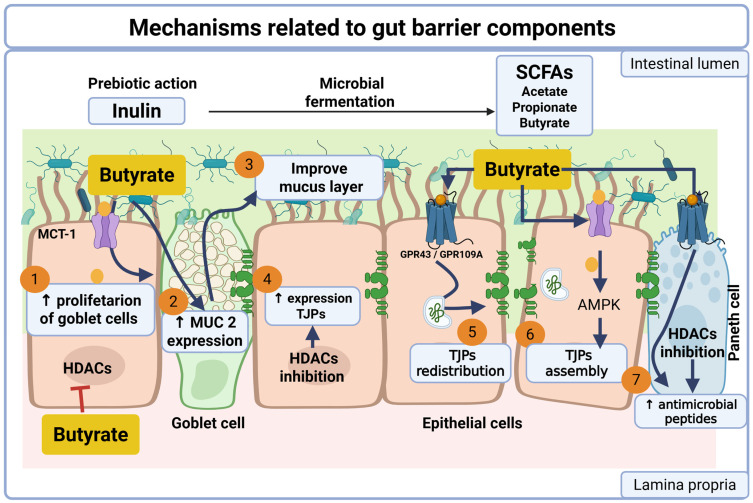
Mechanisms related to gut barrier components. Dietary inulin undergoes microbial fermentation in the colon, leading to the production of short-chain fatty acids (SCFAs), particularly butyrate. Butyrate exerts multiple protective effects on the intestinal barrier. (1) It promotes goblet cell proliferation and (2) increases the expression of MUC2, the main mucin component of the colonic mucus layer, thereby (3) enhancing mucus layer integrity. In epithelial cells, butyrate contributes to barrier function through several mechanisms: (4) epigenetic regulation via inhibition of histone deacetylases (HDACs), resulting in increased expression of tight junction proteins (TJPs); (5) activation of SCFA-sensing G protein-coupled receptors (GPCRs), which promotes TJP relocalization and stabilization at the epithelial junctions; and (6) activation of Adenosine Monophosphate-activated Protein Kinase (AMPK), facilitating tight junction assembly and epithelial integrity. In addition, butyrate can stimulate Paneth cells to increase the production of antimicrobial peptides (7), contributing to antimicrobial defense. Together, these microbiota-dependent mechanisms reinforce the mucus, epithelial, and antimicrobial components of the intestinal barrier. Created in BioRender. Guzman, F. (2026) https://BioRender.com/si1zhdl (accessed on 23 March 2025).

Together, these microbiota-dependent mechanisms reinforce the mucus, epithelial, and antimicrobial components of the intestinal barrier and help explain how microbiota-derived metabolites mediate the protective effects of inulin on intestinal health [64] (Figure 2).

#### 4.3.2. Mechanisms Related to Inflammation

Inulin and FOS can directly modulate inflammatory response, contributing to the preservation of barrier integrity. Although some of these mechanisms may still be influenced by microbial metabolites, experimental evidence from in vitro systems and selected in vivo models supports the existence of host-directed pathways.

At the epithelial level, multiple in vivo studies—including AAA mice, chronic ketamine exposure, chronic kidney disease, and healthy murine models—have consistently shown that inulin increases the expression of TJPs such as ZO-1, Occludin, and Claudin-1 [7,53,54,55,58,61]. This upregulation is closely associated with reduced intestinal permeability and improved epithelial cohesion. In vitro studies further support these findings, as inulin prevented the LPS-induced downregulation of Claudin-1 and Claudin-2 while increasing Occludin expression in Caco-2 cells [7].

Mechanistically, FOSs have been shown to promote tight junction assembly through activation of host signaling pathways. In LPS-primed T84 cells, inulin inhibits signaling pathways of the calcium-sensing receptor (CaSR) and its downstream signaling cascade CaMKKβ–AMPK, which regulates gene expression and epithelial barrier function [59]. Additionally, in Caco-2BBe1 cells infected with EHEC, the barrier-protective effects of inulin and FOS were associated with inhibition of protein kinase C δ (PKCδ) signaling, a pathway associated with tight junction phosphorylation and cytoskeletal remodeling [48].

Beyond tight junction regulation, inulin also contributes to the reinforcement of the antimicrobial barrier. In germ-free C57BL/6 mice with severe acute pancreatitis and in mice fed a Western-style diet, inulin and butyrate promoted Paneth cell proliferation and increased the expression of antimicrobial peptides such as Cryptdin-1, Cryptdin-4, and α-defensin-5 [48,67]. These peptides play a critical role in controlling luminal bacterial populations and maintaining epithelial homeostasis. Furthermore, in healthy C57BL/6 mice, inulin and FOS increased the expression of Fut2, an enzyme involved in epithelial fucosylation, which supports beneficial microbial colonization and contributes to barrier stability [68].

In parallel, inulin and FOS exert significant immunomodulatory effects that help preserve epithelial integrity. Several studies have shown that inulin suppresses key pro-inflammatory pathways activated by xenobiotics. In murine models exposed to PFOA, GenX, or ketamine, inulin inhibited the TLR4–NF-κB–NLRP3 signaling axis, thereby reducing inflammation and pyroptosis in the intestinal mucosa [58,59]. Additionally, inulin can directly interact with innate immune receptors; in PMA-primed T84 cells, inulin’s interaction with TLR2 was associated with inhibition of the TLR2/NF-κB pathway and enhanced barrier protection [46].

Other regulatory pathways of FOS include activation of peroxisome proliferator-activated receptor gamma (PPARγ), which has been linked to reduced production of pro-inflammatory cytokines and increased expression of Peptidoglycan Recognition Protein 3 (PGlyRP3) in Caco-2 cells treated with FOS [69]. Moreover, epigenetic regulation has been proposed as an additional mechanism, as inhibition of HDAC3 has been associated with decreased expression of pro-inflammatory cytokines (TNF-α, IL-1β, IL-6) and polarization of macrophages toward an anti-inflammatory phenotype [53].

Consistent with these effects, inulin and FOS also modulate immune cell dynamics. Experimental models in rodents showed that inulin decreased the number of Ly6C*^hi^* inflammatory cell monocytes, cytotoxic CD8+ T cells, and dendritic cell subsets, along with an increase in regulatory T cells (Treg) [51,61,68]. These changes induced by inulin and FOS were associated with decreased infiltration of inflammatory cells into the intestinal mucosa and improved barrier function.

Finally, modulation of mast cell activity represents an additional mechanism contributing to barrier protection. In a murine model of ovalbumin-induced allergy, inulin reduced mast cell activation and degranulation, thereby limiting the release of mediators that disrupt epithelial integrity [57].

**Figure 3:**
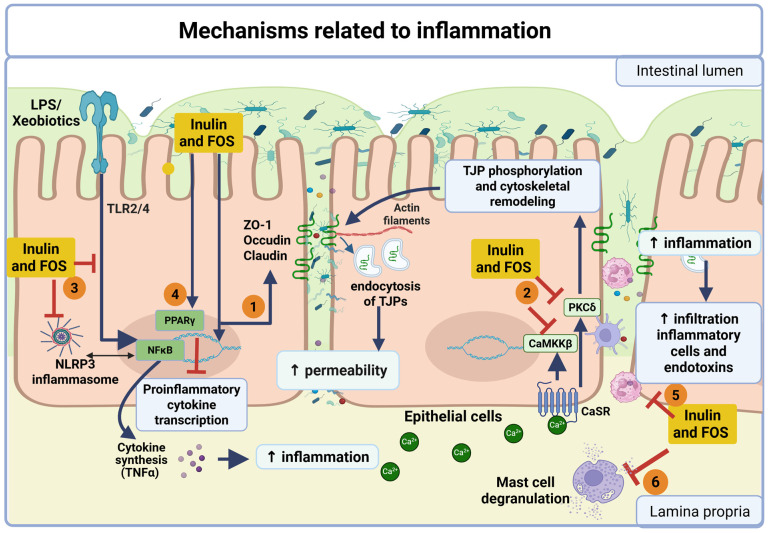
Mechanisms related to inflammation. Inulin and FOS exert protective effects on the intestinal barrier through host-directed mechanisms involving epithelial and immune pathways. (1) Inulin and FOS enhance the expression of TJPs (ZO-1, Occludin, and Claudins), contributing to reduced intestinal permeability. (2) Activation of the calcium-sensing receptor (CaSR) triggers intracellular signaling pathways, including CaMKKβ and PKCδ, promoting tight junction phosphorylation and cytoskeletal remodeling. (3) Inulin and FOS suppress inflammasome activation (NLRP3) and downstream pro-inflammatory signaling, reducing epithelial damage. (4) Modulation of transcriptional pathways, including activation of PPARγ and inhibition of NF-κB, leads to decreased pro-inflammatory cytokine production. (5) These effects are associated with reduced infiltration of inflammatory cells and endotoxins into the lamina propria. (6) Inulin and FOS also inhibit mast cell degranulation, limiting the release of mediators that disrupt epithelial integrity. Collectively, these mechanisms contribute to the maintenance of intestinal barrier function independently or partially independently of microbiota-derived metabolites. Created in BioRender. Guzman, F. (2026) https://BioRender.com/wyrwl9p (accessed on 23 March 2025).

Overall, these findings indicate that inulin exerts protective effects on intestinal permeability through a combination of microbiota-dependent and host-directed mechanisms. While the relative contribution of each pathway may vary depending on the physiological context, the available evidence supports the role of inulin as a functional dietary component capable of preserving epithelial barrier integrity and reducing inflammation. The proposed mechanisms are summarized in Figure 3.

## 5. Effects of Inulin on Intestinal Permeability: Clinical Assays

In clinical assays, inulin’s main mechanism of action is related to the modulation of the gut microbiota, which can improve the integrity of the gut barrier and reduce paracellular permeability. Although inulin’s effects have been poorly studied, some pilot randomized clinical trials indicated that inulin provides beneficial outcomes in healthy populations as well as patients with inflammatory diseases, as described next.

### 5.1. Healthy Patients

In healthy adults exposed to simulated hypobaric hypoxia, inulin supplementation, delivered through snack bars containing fermentable fibers and polyphenols, reduced hypoxia-induced intestinal permeability in the small intestine and proximal colon. These effects were associated with increased *Bifidobacterium* relative abundance, reduced gut microbiota α-diversity and decreased colonic pH, although fecal SCFA concentrations were unchanged. Notably, supplementation was linked to increased intestinal symptoms and altitude sickness during hypoxia exposure [70].

Similarly, in a randomized, double-blind crossover study in healthy young adults, consumption of inulin-enriched pasta increased circulating glucagon-like peptide 2 (GLP-2) levels and significantly reduced intestinal permeability, as measured by urinary lactulose recovery and serum zonulin, without affecting urinary mannitol excretion. These findings suggested that dietary inulin preserves the functioning of the intestinal mucosal barrier under healthy conditions and may contribute to the prevention of gastrointestinal and metabolic disorders [71].

### 5.2. Inflammatory Bowel Diseases (IBD)

The effects of inulin on intestinal permeability have been primarily explored in the context of inflammatory bowel disease (IBD), particularly Crohn’s disease (CD), where alterations in gut microbiota composition and epithelial barrier dysfunction play central pathogenic roles. However, clinical evidence indicates that inulin supplementation may exert both beneficial and potentially adverse effects, depending on disease activity, host microbiota composition, and fermentation tolerance. In patients with inactive CD and siblings at increased risk for disease development, a combined inulin/FOS preparation (15 g/day for 3 weeks) increased fecal *Bifidobacterium* (*B.*) *longum* and reduced intestinal permeability, measured by the urinary lactulose–rhamnose ratio. These findings suggest a prebiotic-mediated improvement in barrier function associated with microbiota modulation. Nevertheless, no significant changes were observed in calprotectin levels, indicating that improvements in permeability did not translate into measurable reductions in intestinal inflammation during the intervention period [72]. Importantly, interindividual variability was observed, as one participant exhibited increased inflammatory markers following supplementation, highlighting that fermentable fibers such as inulin may not be universally tolerated in IBD patients. Excessive fermentation and gas production have been proposed as mechanisms that could exacerbate gastrointestinal symptoms in susceptible individuals, particularly during active disease phases.

### 5.3. Celiac Disease

Regarding celiac disease, the intestinal permeability was recorded following a gluten-free diet (GFD) with prebiotic FOS-enriched inulin (10 g per day) for 12 weeks in children with celiac disease. Results of this study showed that prebiotic supplementation did not have a significant effect on intestinal permeability evaluated by a plasma zonulin test. However, a moderate positive correlation was observed between the excretion of lactulose: mannitol, indicating a recovery of intestinal permeability in celiac disease. FOS/inulin intake in patients normalized the elevated levels of lactulose: mannitol and calprotectin ratio. Despite these findings, the small number of patients limits the interpretation of these results [73].

### 5.4. Nutritional Support

In patients who required the nutritional support of a liquid enteral diet, inulin (30–35 g/d) was added as dietary fiber to fiber-free enteral nutrition for 1 week. Inulin increases bacterial fermentation. However, no changes in fecal SCFAs or intestinal permeability, assessed by 51Cr-EDTA absorption test, were seen [74]. The lack of effect of inulin on intestinal permeability could be attributed to the short period of administration. Furthermore, the wide array of diseases (ulcerative colitis, mental anorexia and pseudobulbar paralysis) limited the interpretation of results of this study.

Although human trials have evidenced the effect of inulin on intestinal permeability, in another trial in African children with severe malnutrition, this beneficial effect of inulin was not found [75]. In this trial, no significant effects of inulin on intestinal permeability measured with lactulose: mannitol ratio were found [75].

With respect to liver disease, the effects of inulin in patients with metabolic-associated fatty liver disease (MAFLD) in combination with type 2 diabetes mellitus (T2D) were evaluated. In this study, inulin was provided in lyophilized form containing metformin, rifaximin and a concentration of Jerusalem artichoke (6 g per sachet) at doses of 1 sachet twice a day for 6 months. This combined therapy led to a reduction in subclinical inflammation and a decrease in serum zonulin [76]. These findings evidenced the impact of inulin on gut permeability in MAFLD and TD2 patients.

### 5.5. Liver Diseases

A study on non-alcoholic steatohepatitis (NASH) patients tested the impact of intake of a synbiotic, composed of *Lactobacillus* (*L.*) *reuteri*, guar gum and inulin as prebiotics, for 3 months, on intestinal permeability. Evidence from this study showed that this synbiotic reduced steatosis. The synbiotic did not improve the intestinal permeability or lead to a reduction in LPS levels; however, the symbiotic supplementation did not provide concluding data associated with improved intestinal permeability in these patients [77].

Another study in pediatric patients with type 1 diabetes (T1D) evaluated FOS-enriched inulin (8 g orally/day for 3 or 6 months). In this study, intestinal permeability was evaluated by the lactulose: mannitol ratio. Evidence showed that prebiotics significantly increased the relative abundance of *Bifidobacteria* and had an unsignificant effect on intestinal permeability as compared to the placebo group. Prebiotics had no impact on diabetic ketoacidosis or severe hypoglycemia. These results suggested that prebiotics did not have an impact on intestinal permeability [78,79].

### 5.6. Metabolic Diseases

Continuing with metabolic diseases, a synbiotic preparation containing *L. paracasei*, *B. longum*, and *B. breve* as probiotics and 5 g of inulin and 5 g of FOS as prebiotics was administered for 12 weeks in Thai subjects with obesity. The findings showed that the individuals fed the synbiotic preparation had a significant decrease in anthropometric, biochemical and inflammatory parameters; importantly, the synbiotic preparation caused a significant reduction in the intestinal permeability tested by zonulin assay. This study suggests that synbiotic supplementation may have a beneficial effect on the intestinal permeability and body weight in patients with obesity [80].

In another study conducted in women with obesity and mild to moderate depression, the effect of inulin alone at doses of 10 g/day for 8 weeks was evaluated. In this trial, patients received a stable antidepressant regimen for ≥6 months prior to the study and maltodextrin was used as a placebo. Results display that inulin did not show significant effects on anthropometric measures, depressive symptoms, gut permeability (evaluated by zonulin test), endotoxemia (serum LPS) and inflammatory biomarkers (serum TNF-α, IL-10, MCP-1, TLR-4 and hs-CRP). However, the lack of significant results could be attributed to short-term nature of the study [81].

In type 2 diabetes (T2D), another metabolic condition, the impact of inulin on intestinal permeability was evaluated. In this study inulin was administered at dose of 10 g per day, for 6 weeks. The results showed that inulin significantly reduced fasting insulin when comparing baseline and week 6 within the inulin group. However, inulin intake did not affect intestinal permeability, evaluated in the gastro-duodenal region by the sucrose: mannitol test, in the small intestine by lactulose: mannitol assay, or in the colon using the sucrose: mannitol test and LPS levels. Results of this pilot study suggest that inulin does not improve the intestinal permeability in adults at risk for T2D. Further studies on a larger scale in this population are needed to address the impact of inulin in gastrointestinal permeability [82].

### 5.7. Coronary Diseases

In regard to coronary heart disease, a synbiotic preparation containing prebiotic inulin and *L. rhamnosus* as probiotic at a dose of 15 mg/day for 60 days was evaluated in a randomized, double-blind trial in patients with coronary artery diseases. Results of this study showed that the synbiotic decreased both the intestinal permeability, evaluated by serum zonulin, and systemic inflammation, evidenced by reduced serum levels of serum IL-6, and TLR-4 and increased serum total antioxidant capacity. In addition, the increased cannabinoid receptor (CB2) mRNA expression correlated with the decreased serum levels of LPS; also, *Firmicutes*/*Bacteroidetes* (F/B) ratio decreased, suggesting an improved health status; F/B ratio increase is a parameter that reflects heart issues and metabolic disorders. These results evidence that the synbiotic preparation enhanced the regulatory effect on intestinal permeability and inflammation through the microbiota composition and CB2 receptor expression [83].

Finally, migraine is another pathological condition in which inulin has been evaluated in a randomized, double-blind, placebo-controlled clinical trial. In this study inulin was administered alone at a dose of 10 g/day for 12 weeks in women with migraine. In this study the effects of inulin on the intestinal permeability, evaluated by zonulin and other serum markers of inflammation and oxidative status, were evaluated. No conclusive effects of inulin on intestinal permeability, inflammation or migraine severity were observed. In spite of these findings, the impact of inulin in migraine and intestinal permeability can be addressed in studies using other schemes of inulin supplementation to identify mechanistic action [84].

Overall, clinical evidence evaluating inulin and intestinal permeability remains inconclusive and highly context-dependent. Positive outcomes are more frequently reported in studies involving inflammatory or metabolically compromised populations, whereas trials in healthy individuals or metabolic disorders often show no effects.

Importantly, variability in dose, chain length (native inulin vs. FOS-enriched preparations), treatment duration, co-interventions, and permeability assessment methods represents a major source of heterogeneity across studies. Short interventions (<4 weeks) and mixed formulations appear less likely to demonstrate consistent barrier improvements, although systematic dose–response relationships cannot yet be established due to insufficient comparative trials.

Several mechanistic pathways—including microbiota-derived metabolites and downstream signaling, potentially involving inflammatory or metabolic regulators, have been proposed. Direct clinical evidence linking inulin supplementation to specific molecular pathways in humans remains limited, and these mechanisms should therefore be considered hypothetical or extrapolated primarily from experimental models.

Consequently, current human data support a potential but not definitive role for inulin as a modulator of intestinal permeability, underscoring the need for larger, well-controlled clinical trials with standardized formulations, dosing strategies, and validated permeability endpoints.

## 6. Conclusions

Up to now, dysfunctional intestinal permeability, causing “leaky gut”, is regarded as a hallmark of several inflammatory chronic conditions like colitis, Crohn’s disease, celiac disease, and irritable bowel syndrome, with outcomes in extraintestinal clinical entities like non-alcoholic fatty liver, type 1 and type 2 diabetes, depression and so on [85]. Therefore, inulin has been a focus of interest given its beneficial outcome in a wide array of diseases with dysfunctional gut barrier permeability [39]. Experimental assays have described underlying mechanisms and provide compelling evidence that support the effect of inulin on the regulation of intestinal inflammatory dysfunctions through fermentative-microbiota derivatives such as butyrate or by interaction with innate components in the epithelial monolayer [86]. Given its regulatory effects on gut barrier components, inulin has been included as a therapeutic component in oral drug formulations [87]; moreover, due to its biocompatibility, gastro-intestinal stability and selective biodegradability in the colon, potential translational challenges entail the application of inulin as a vehicle for inulin-based nanoparticles targeted to colon dysfunctions like ulcerative colitis, which affects intestinal permeability [88,89,90]. Oral inulin-based formulations are intended to increase the drug-action target in the colon, and to enhance the drug-therapeutic efficiency by reducing the dose, tolerance and potential side effects after being administered orally a as preferred route of administration compared to invasive injectable routes [90]. Currently, several gaps in knowledge persist. These include the differential impact of molecular size on gut permeability for both inulin and FOS, the mechanisms underlying their regulation of permeability, and the translational impact of these findings in human studies, which are currently limited.

Although inulin is innocuous fiber, the lack of microbiota-derived enzymes for the breakdown of inulin result in undesirable side effects such as intolerance [91,92] at the colon level; undegraded fructans favor the outflow of water to the lumen, which can result in bloating and diarrhea [92].

According to the above, inulin may provide beneficial effects for gut barrier permeability, and future studies are needed to test its safety and reduce its potential risks and hazards.

## Figures and Tables

**Table 1:** Effect of inulin treatment on intestinal permeability and integrity in vitro.

Model	Treatment	Intestinal Integrity/Permeability Assessment	Observed Effect	Ref.
Caco-2 cell monolayers challenged with DON.	Inulin (0.5%, 1% and 2% *w*/*v* in culture medium), pre-incubation for 24 h.	TEER measurement and LY flux assay.	Inulin has no significant effect on the prevention of DON-induced barrier dysfunction.	[43]
Monolayers of human Caco-2 cells.	Inulin (0.5 mg/mL in culture medium) for 6 h.	TEER measurement.	Inulin has no significant effect on the modulation of intestinal integrity.	[44]
Caco-2 cell, untreated.	FOS (100 mmol/L in culture medium), pre-incubation for 3 h.	TEER measurement, transepithelial net calcium transport across and LY flux assay.	FOS significantly increased intestinal permeability.	[45]
T84 intestinal epithelial cells challenged with phorbol myristate acetate.	FOS and inulin (100 mg/L *w*/*v* in culture medium), pre-incubation for 24 h.	TEER measurement.	FOS significantly prevent PMA-induced loss of intestinal integrity, while inulin had no significant effect.	[46]
Monolayers of human T84 epithelial cells, challenged with A23187, DON or PMA	Inulin (10 mg/mL in culture medium), pre-incubation for 24 h.	TEER measurement.	Inulin significantly prevent A23187- and DON-induced intestinal disruption. Inulin has no significant effect on phorbol myristate-induced intestinal disruption.	[47]
Caco-2 cell monolayers infected with enterohemorrhagic *E. coli O157:H7* (EHEC).	Inulin (10% *w*/*v* in culture medium), pre-incubation for 16 h.	TEER measurement.	Inulin significantly enhances intestinal integrity under basal conditions. Inulin has no significant effect on permeability in EHEC-infected cells.	[49]
Human intestinal Caco-2 cells challenged with LPS.	Inulin (2% *w*/*v* in culture medium), pre-incubation for 24 h.	FITC-Dextran flux assay and expression of Claudin-1, Claudin-2 and Occludin.	Inulin significantly attenuated LPS-induced increase in intestinal permeability and prevented alterations in TJP expression.	[7]
Caco-2Bbe1 cell infected with EHEC.	Inulin and FOS (1–15% *w*/*v* in culture medium), pre-incubation for 16 h.	TEER measurement, FITC-Dextran flux assay and ZO-1 mRNA and protein expression.	Inulin significantly enhances intestinal integrity in basal conditions. Inulin and FOS prevented EHEC-induced loss of intestinal integrity and increased permeability. FOS upregulated ZO-1 expression in EHEC-challenged cells.	[48]

**Table 2:** Effect of inulin treatment on intestinal permeability and integrity in vivo.

Model	Treatment	Intestinal Integrity/Permeability Assessment	Observed Effect	Ref.
Female Sprague-Dawley rats (Healthy).	Inulin (5% *w*/*w* in chow diet), for 4 weeks.	Sucrose and lactulose: mannitol ratio test.	Inulin has no significant effect on intestinal integrity.	[51]
Female BALB/c mice (Healthy).	Inulin (10% *w*/*w* chow diet), for 2 weeks.	FITC-dextran flux assay.	Inulin significantly reduced intestinal permeability under basal conditions.	[50]
Male Kunming mice (Healthy).	Inulin (2.5 g/kg body weight), orally, for 4 weeks.	Expression of Occludin, ZO-1 and Mucin-2.	Inulin significantly increased expression of TJPs and Mucin-2.	[55]
Male C57BL/6 mice on a high-fat diet (40% fat/Kcal).	Inulin (6% *w*/*w* in high-fat diet), for 6 weeks.	Plasma LPS-binding protein (LBP) measurement and FITC-dextran flux assay (ex vivo).	Inulin significantly prevented high-fat-diet-induced increase in intestinal permeability.	[52]
Male C57BL/6 mice on a high-fat diet (45% fat/Kcal).	Inulin (10% *w*/*w* in high-fat diet), for 4 weeks.	Ussing chamber system (ex vivo).	Inulin significantly enhanced intestinal integrity in mice on a high-fat diet.	[56]
Male germ-free C57BL/6 mice with severe acute pancreatitis induced by high-fat diet.	Inulin (37 g per 1000 Kcal in high-fat diet), for 4 weeks.	Plasma LPS measurement, D-lac assay and expression of ZO-1, Occludin, Claudin-1, and E-Cadherin.	Inulin significantly prevented high-fat-diet-induced increased in intestinal permeability. Inulin significantly increased TJP expression and promoted Paneth cell proliferation and antimicrobial peptide production.	[53]
Female C57BL/6 mice infected with *Citrobacter rodentium*.	Inulin and FOS (10% *w*/*v* in drinking water), for 10 days.	FITC-dextran assay.	Inulin and FOS have no significant effect in preventing infection-induced increases in intestinal permeability.	[49]
Female BALB/c mice in a food allergy model	Inulin (20, 50 and 80 mg/day orally), for 20 days.	Serum LPS measurement and expression of Occludin, Claudin and ZO-1.	Only medium doses of inulin significantly prevented the food allergy-induced increase in intestinal permeability and the associated decrease in TJP expression.	[57]
Male C57/BL6 mice with chronic ketamine treatment.	Inulin (2 g/kg dissolved in drinking water), for 6 weeks.	Serum and colonic LPS measurement and expression of ZO-1 and Occludin.	Inulin significantly prevented the ketamine-induced increased in intestinal permeability and the associated decrease in TJP expression.	[58]
C57BL/6J mouse pups perinatally exposed to toxins (PFOA or GenX).	Inulin (5 g/kg body weight/day), orally, during pregnancy and lactation.	FITC-dextran assay.	Inulin significantly prevented PFOA- and GenX-induced increases in intestinal permeability and suppressed the TLR4/NF-κB/NLRP3 pathway and pyroptosis.	[59]
Male C57BL/6 mice under chronic unpredictable mild stress model.	Inulin (0.037 g of inulin/Kcal chow diet), for 9 weeks.	It decreased the LPS level in serum.	Inulin significantly prevented the stress-induced increase in intestinal permeability and inhibited the TLR4/MyD88/NF-κB pathway in the brain and gut.	[60]
Male BTBR mice (autism spectrum disorder mouse model).	Oligofructose-enriched inulin (10% *w*/*w* in chow diet), for 3 weeks.	FITC-Dextran flux assay.	Oligofructose-enriched inulin has no significant effect on intestinal permeability.	[62]
Male C57BL/6 mice with abdominal aortic aneurysm.	Inulin (15% *w*/*w* in chow diet), for 6 weeks.	FITC-Dextran flux assay and expression of ZO-1, Occludin, Mucin-2 and Cadherin-1.	Inulin significantly prevented aneurysm-induced increased in intestinal permeability and the associated decrease in TJP expression.	[61]
Male Sprague-Dawley rats with adenine-induced chronic kidney disease.	Oligofructose-enriched inulin (5 g/kg body weight in their drinking water per day), for 4 weeks.	Serum p-cresol sulfate (PCS) levels and expression of Occludin and Claudin-1.	Oligofructose-enriched inulin significantly prevented adenine-induced increase in intestinal permeability and the associated decrease in TJP expression.	[54]

## Data Availability

No new data were created or analyzed in this study.
